# A Narrative Review of the Neurological Manifestations of Human Adenosine Deaminase 2 Deficiency

**DOI:** 10.1007/s10875-023-01555-y

**Published:** 2023-08-07

**Authors:** Mariia Dzhus, Lisa Ehlers, Marjon Wouters, Katrien Jansen, Rik Schrijvers, Lien De Somer, Steven Vanderschueren, Marco Baggio, Leen Moens, Benjamin Verhaaren, Rik Lories, Giorgia Bucciol, Isabelle Meyts

**Affiliations:** 1 Department of Microbiology, Immunology and Transplantation, Inborn Errors of Immunity, KU Leuven, Leuven, Belgium; 2 Department of Development and Regeneration, Department of Pediatrics, University Hospitals Leuven and KU Leuven, Leuven, Belgium; 3 Department of General Internal Medicine–Allergy and Clinical Immunology, Allergy and Clinical Immunology Research Group, Department of Microbiology, Immunology and Transplantation, University Hospitals Leuven and KU Leuven, Leuven, Belgium; 4 Department of Pediatric Rheumatology, Laboratory of Immunobiology, Rega Institute, European Reference Network for Rare Immunodeficiency, Autoinflammatory and Autoimmune Diseases, University Hospital Leuven and KU Leuven, Leuven, Belgium; 5 Department of General Internal Medicine, European Reference Network for Rare Immunodeficiency, Autoinflammatory and Autoimmune Diseases, Department of Microbiology, Immunology and Transplantation, University Hospitals Leuven and KU Leuven, Leuven, Belgium; 6 Department of Radiology, University Hospitals Leuven, Leuven, Belgium; 7 Department of Development and Regeneration, Skeletal Biology and Engineering Research Centre, Division of Rheumatology, University Hospitals Leuven and KU Leuven, Leuven, Belgium; 8 Department of Microbiology, Immunology and Transplantation, Inborn Errors of Immunity, Department of Pediatrics, University Hospitals Leuven and KU Leuven, Leuven, Belgium; 9 Department of Microbiology, Immunology and Transplantation, Inborn Errors of Immunity, Department of Pediatrics, European Reference Network for Rare Immunodeficiency, Autoinflammatory and Autoimmune Diseases, University Hospitals Leuven and KU Leuven, Leuven, Belgium

**Keywords:** Adenosine deaminase 2, DADA2, childhood strokes, vasculitis

## Abstract

Deficiency of human adenosine deaminase type 2 (DADA2) is a complex systemic autoinflammatory disorder characterized by vasculopathy, immune dysregulation, and hematologic abnormalities. The most notable neurological manifestations of DADA2 are strokes that can manifest with various neurological symptoms and are potentially fatal. However, neurological presentations can be diverse. We here present a review of the neurological manifestations of DADA2 to increase clinical awareness of DADA2 as the underlying diagnosis. We reviewed all published cases of DADA2 from 1 January 2014 until 19 July 2022 found via PubMed. A total of 129 articles describing the clinical features of DADA2 were included in the analysis. Six hundred twenty-eight patients diagnosed with DADA2 were included in the review. 50.3% of patients had at least signs of one reported neurological event, which was the initial or sole manifestation in 5.7% and 0.6%, respectively. 77.5% of patients with neurological manifestations had at least signs of one cerebrovascular accident, with lacunar strokes being the most common and 35.9% of them having multiple stroke episodes. There is a remarkable predilection for the brain stem and deep gray matter, with 37.3% and 41.6% of ischemic strokes, respectively. Other neurological involvement included neuropathies, focal neurological deficits, ophthalmological findings, convulsions, and headaches. In summary, neurological manifestations affect a significant proportion of patients with DADA2, and the phenotype is broad. Neurological manifestations can be the first and single manifestation of DADA2. Therefore, stroke, encephalitis, posterior reversible encephalopathy syndrome, mononeuropathy and polyneuropathy, and Behçet’s disease-like presentations should prompt the neurologist to exclude DADA2, especially but not only in childhood.

**Supplementary Information:**

The online version contains supplementary material available at 10.1007/s10875-023-01555-y.

## Introduction

Deficiency of human adenosine deaminase type 2 (DADA2) was first described in 2014 by two independent groups as a condition characterized by fever, polyarteritis nodosa (PAN), livedo racemosa, early-onset stroke, liver disease, and mild immunodeficiency [1, 2]. Subsequently, the phenotype expanded to include additional hemato-immunological manifestations such as cytopenias, bone marrow failure, and hemophagocytosis [3–5]. The underlying cause of DADA2 is biallelic (homozygous or compound heterozygous) loss of function mutations in adenosine deaminase type 2 (ADA2), formerly known as Cat Eye Syndrome Chromosome Region 1 (CECR1) [1, 2].

DADA2 usually presents in childhood, with an average age of onset of 5 to 7 years; approximately 25% of patients have the onset of the symptoms before the age of 1 year and 77% by the age of 10 years [4, 6]. However, adult-onset has also been described [1, 2, 7–9]. In some cases, DADA2 can be misdiagnosed as PAN. Caorsi et al. described DADA2 in 15 (31%) out of 48 European children with early-onset PAN [10]. In adult patients with PAN, biallelic pathogenic ADA2 variants were detected in 4 out of 108 cases (3.4%) [11]. The incidence of DADA2 has been estimated to be around 1 in 222,000 individuals worldwide [12], with a mortality of 8% [4], mainly in childhood.

The pathophysiology of DADA2 remains incompletely understood [1, 2]. ADA2 is mainly but not exclusively expressed by myeloid cells, and its deficiency leads to impairment of endothelial integrity and the development of perivascular inflammation. Tumor necrosis factor (TNF) is a key cytokine involved in the pathological process but increased type I and II interferon (IFN) signaling have also been described [13, 14]. The monocyte differentiation in patients with DADA2 has been reported to be skewed, resulting in a decreased number of anti-inflammatory M2 macrophages and increased proinflammatory M1 macrophages [15]. ADA2 may play a role in the activation of neutrophils; thus, in ADA2-deficient patients, hyperactivation of neutrophils may cause endothelial damage [16]. Additionally, patients with DADA2 have been described to display an increased production of neutrophil extracellular traps (NETs) and subsequently increased TNF production [17].

Most likely, a dysregulation of the homeostasis of the vascular endothelium in the presence of proinflammatory macrophages leads to vessel stenosis, aneurysm formation, and perforation of vessels [6]. This process primarily affects small and medium vessels and manifests in various locations, including the skin, cranial and peripheral nerves, kidneys, intestine, and even testes [18]. Signs and symptoms of DADA2 range from non-specific signs, such as fever, and weight loss, to specific findings resembling PAN, such as nodular vasculitis, livedo racemosa, ulcers, abdominal pain, bowel perforation, and portal or arterial hypertension. Since the histopathological findings of vasculitis are indistinguishable between PAN and DADA2, the latter is sometimes classified as a subtype of “classic” PAN [1], representing a more aggressive variant.

Notably, neurological complications are common in DADA2 [19], with strokes being a prominent feature that can present with various neurological symptoms and carry a high risk of fatality [1, 2, 10, 20]. Other neurological findings or initial alternative diagnosis include transient ischemic attacks (TIAs) with insignificant magnetic resonance imaging (MRI) findings, posterior reversible encephalopathy syndrome (PRES)-like encephalopathies, sensorineural hearing loss, ophthalmologic abnormalities, and peripheral and cranial neuropathies [21, 22]. Neurological symptoms can precede other manifestations in the course of DADA2 or be a sole presenting feature [4, 6, 23, 24]. Given the significant neurological impact of DADA2 and the need for increased clinical awareness, this paper aims to provide a comprehensive review of the neurological manifestations associated with this condition. By summarizing the relevant cases reported in the current scientific literature, we aim to enhance understanding and facilitate early recognition and management of DADA2-related neurological complications.

## Methods

**Fig. 1:**
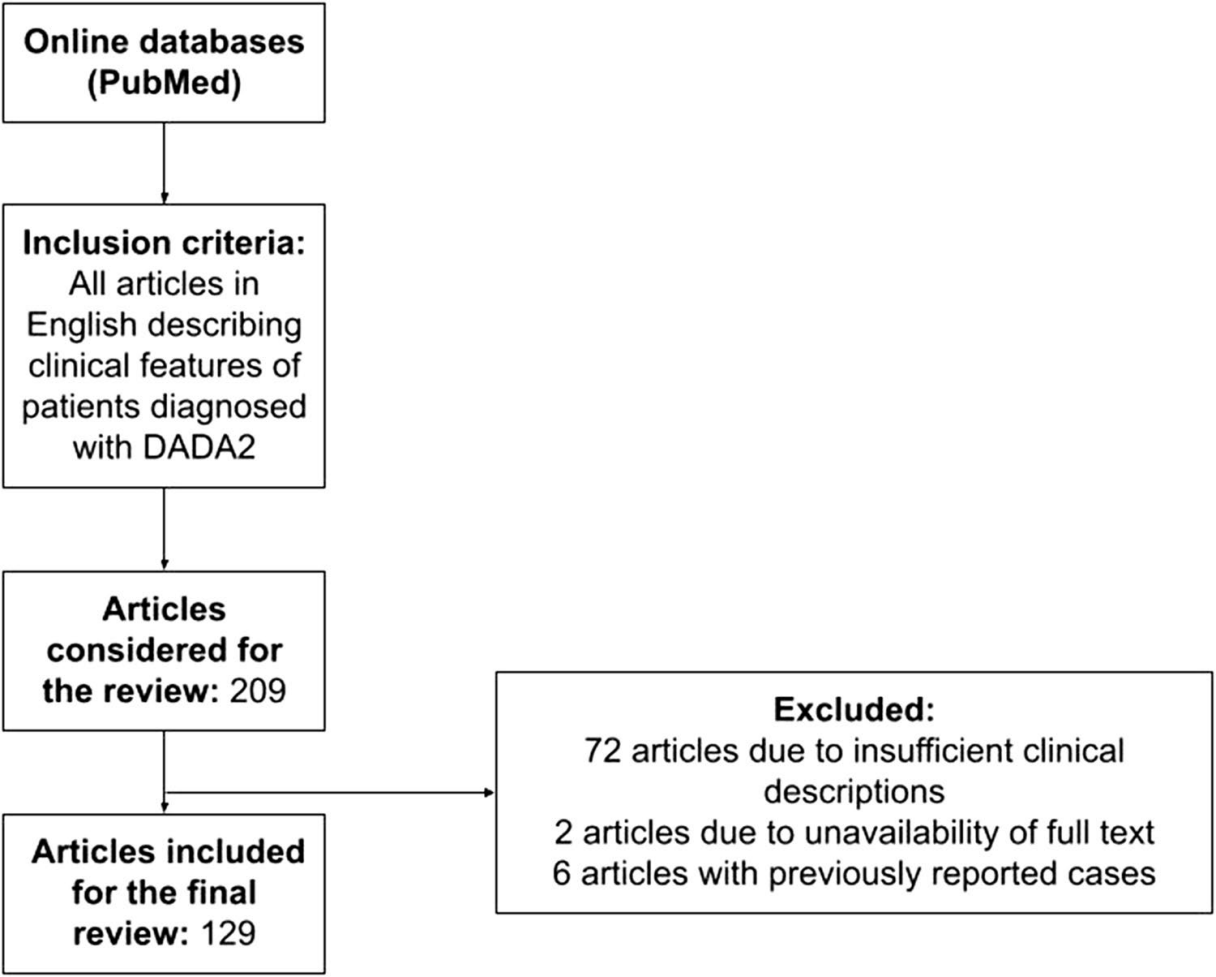
Flowchart of literature search

We reviewed the available data on patients diagnosed with DADA2 published between 1 January 2014 and 19 July 2022. The search was conducted manually via PubMed using the following keywords: “ADA2 deficiency OR adenosine deaminase 2 deficiency OR DADA2.” After all articles describing the clinical features of DADA2 were included in the analysis. Articles that were not in English were not included in the study. Papers containing insufficient clinical descriptions of patients were excluded [3, 4, 6, 11, 12, 14, 17, 19, 25–87]. This was evaluated by full-text screening. In case of duplication of cases, only the most recent report or the report with more complete clinical description was included [88–93]. Two articles were not included because of unavailability of the full text [94, 95] (Fig. 1).

## Results

A total of 628 patients diagnosed with DADA2 were included in the review to analyze the demographics, genotypes, and clinical characteristics.

### Demographics

There were 221 (46.4% of patients with known sex) female patients, 255 (53.6%) male patients, and 152 patients whose sex was not specified.

Of 274 patients with reported ethnicity (43.6%), 137 were Caucasian (50%), 43 were South Asian (15.7%), 27 were East Asian (9.9%), 43 came from the Middle East (15.7%), 9 North African (3.3%), 9 Latin American (3.3%), 2 Jewish (0.7%), 2 African/Caucasian (0.7%), 1 African (0.4%), and 1 African American (0.4%).

The mean age of disease onset for 425 patients (67.7%) for whom it was reported was seven years. Thirty-three reported patients (7.8%) had an age of onset ranging between 18 and 59 [2, 9, 20, 96–105]. In 20 of these, the adult presentation was with stroke or another neurological manifestation.

### Genetics

The notations of the patients’ mutations in the ADA2 gene were standardized according to the NM_001282225.2 transcript.

**Fig. 2:**
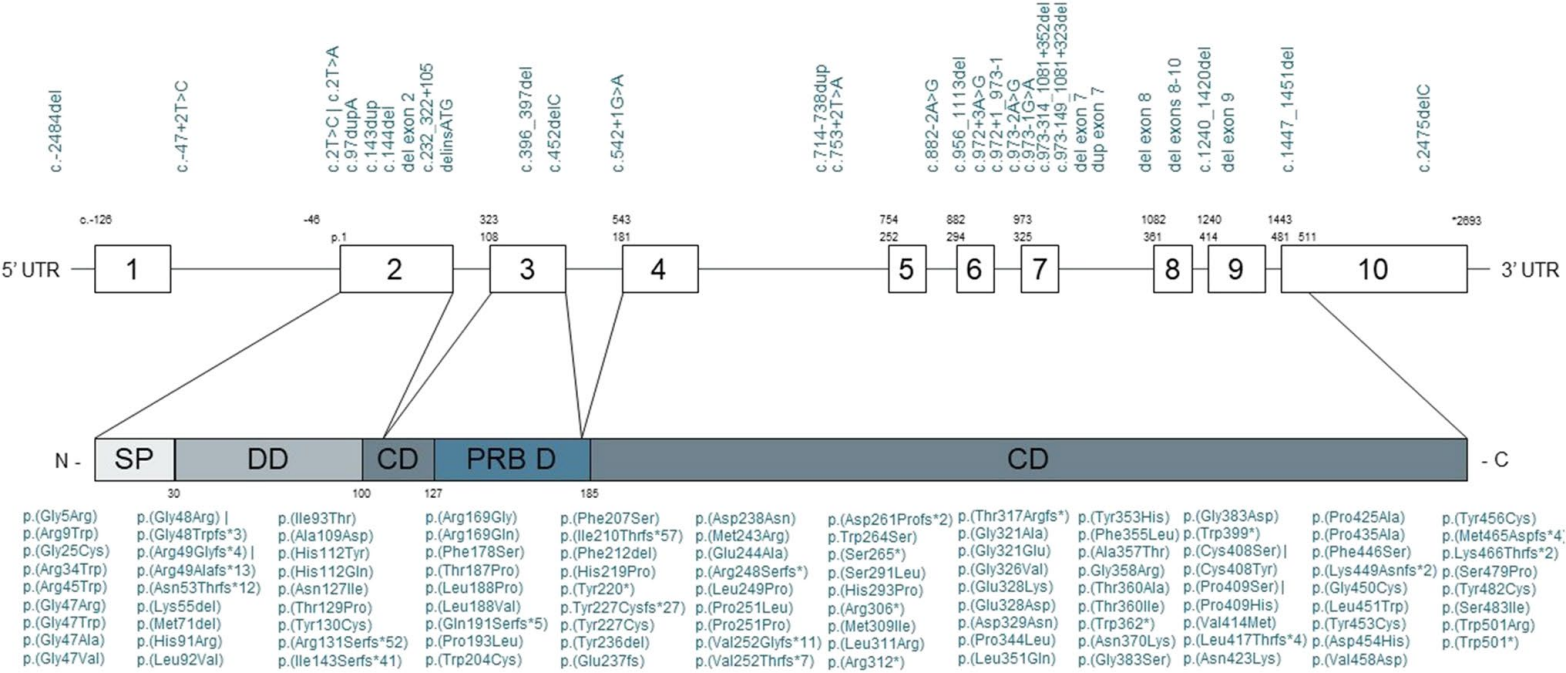
Pathogenic variants of ADA2. Genomic sequence with exonic regions indicated by boxes and the corresponding protein with domains indicated below. Reported pathogenic variants are indicated above. SP, signal peptide; DD, dimerization domain; CD, catalytic domain; PRB D, putative receptor binding domain

The pathogenic variants were reported for 495 patients (78.8%). Two hundred seventy-seven patients had a homozygous variant, 205 compound heterozygous, 12 patients had one heterozygous variant with an unknown second mutation, and one patient had both homozygous and heterozygous deletions. There were 82 missense mutations, 28 frameshift mutations, 9 splice site mutations, 7 nonsense mutations, 3 duplications, 18 deletions, 2 start loss mutations, 1 deletion-insertion mutation, and 1 unspecified intronic mutation. Figure 2 and Supplementary Table S1 present the genetic characteristics in detail.

### Clinical Characteristics

#### Neurological Manifestations

**Fig. 3:**
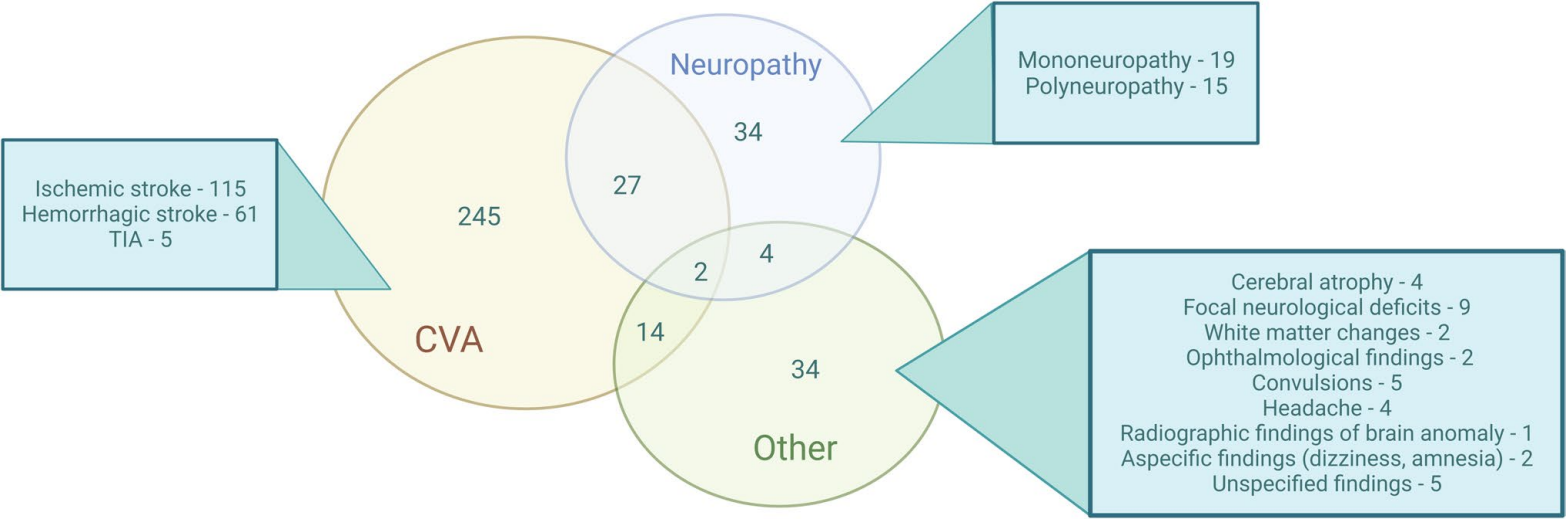
The spectrum of neurological symptoms in patients with DADA2 (*n*=316). CVA, cerebrovascular accident; TIA, transient ischemic attack

Among the reviewed patients, 316 (50.3%) had at least one reported neurological event throughout the course of the disease. The mean age of onset of neurological manifestation was 7.2 years (range: 0–36 years). A total of 245 patients (77.5% of all neurological manifestations) had at least one cerebrovascular accident (CVA), including 115 ischemic, 61 hemorrhagic strokes, and five transient ischemic attacks; 88 patients (35.9%) had multiple stroke episodes. Thirty-four patients presented with neuropathy (19 patients with mononeuropathies and 15 patients with polyneuropathies). Other neurological involvements included focal neurological deficits (such as cranial nerve paralysis, decreased/increased tendon reflexes, upper motor neuron signs, numbness, extremity weakness, sensory loss in the lower extremity) (*n*=9), ophthalmological findings (such as strabismus, loss of vision) (*n*=2), convulsions (*n*=5), headache, including migraines (*n*=4), and aspecific findings like dizziness and amnesia (*n*=2). In addition, cerebral atrophy (*n*=4), white matter changes (*n*=2), radiographic findings of brain anomaly (*n*=1), and five patients with unspecified neurological findings were reported. The spectrum of neurological symptoms in patients with DADA2 is shown in Fig. 3.

For 18 patients (5.7% of patients with neurological involvement), a neurological event represented the initial presentation of DADA2 [2, 13, 21, 23, 24, E6–E14]; for two of them [2, 13], it was the sole manifestation of the disease, as reported in the literature. However, the majority of patients initially presented with a variety of hematological, immunodeficient, and vascular manifestations, such as recurrent fever, weight loss, recurrent upper airway infection, livedo racemosa or reticularis, skin and oral aphthous ulcers, Raynaud phenomenon, arterial hypertension, myocarditis, abdominal pain, diarrhea, hepatosplenomegaly, myalgia, arthritis, alopecia, hypogammaglobulinemia, cytopenia, testicular, mesenteric, pancreatic, splenic, and renal infarcts. It is worth noting that the existing literature and awareness of DADA2 largely stem from rheumatology and immunology studies, potentially resulting in skewed representation of neurological manifestations.

Twenty-two patients (3.5% of all the reviewed patients) had neurocognitive disorders [1, 8, 13, 21, 97, E9, E15–E21]. These included isolated cognitive impairment, isolated memory disturbance, autism spectrum disorder (ASD), attention deficit hyperactivity disorder (ADHD), isolated persistent irritability, and isolated difficulties in concentration. Most of the patients were reported to have only one type of neurocognitive disorders; only those diagnosed with ASD also had ADHD, and one patient with memory disturbance also had difficulties in concentration. The age of the disease onset of the patients with neurocognitive disorders ranges from 0 to 14 years, with the mean age of 4.3 years.

MRI findings of 146 patients were available (Table 1) [1, 2, 10, 13, 16, 20–24, 96, E1, E4–E7, E9–E11, E13–E15, E17–E19, E21–E51]. Other diagnostic reports for neurological involvement included computer tomography [2, E4, E52–E54], angiographic investigation (reveals arterial stenosis, aneurysms) [1, 2], nerve conduction study, and needle electromyography [E55] (both considered to serve as non-specific diagnostic tools of neuropathies in DADA2). According to the review of neuroimaging results, the most common CNS lesion during DADA2 is ischemic stroke, with most common localizations in the brain stem, thalamus, basal ganglia, internal capsule, and other deep cerebral structures, representing the pathological involvement of deep perforating arteries. Lacunar stroke cases comprise 62.7% of the available MRI findings, of which 90.2% were located in the deep gray matter or brain stem. Vascular wall enhancement of small perforating arteries has also been reported [E32], but may be under-represented because of the absence of vessel-wall-imaging sequences in studies. We note that the described imaging findings are based on MRI, which is the preferred imaging modality due to its ability to detect acute ischemic stroke using diffusion-weighted sequences and its suitability for comprehensive evaluation of intracranial manifestations in children due to the absence of ionizing radiation.

**Table 1:** MRI findings in patients with DADA2 — quantified as number of MRI’s reporting this trait. *PRES*, posterior reversible encephalopathy syndrome. *May represent an under-representation in the study population, because of limited availability of vessel wall imaging, contrast enhanced studies, angiographic imaging (MRA/CTA/DSA), and susceptibility weighted imaging. ^§^Presumed chronic ischemic white matter lesions without lacune formation

MRI findings			№	%
Lacunar cerebral infarcts			183	62.7
Brain stem №=78 (26.7%)	Midbrain		32	11.0
	Pons		20	6.8
	Medulla oblongata		3	1.0
	Other (pontomesencephalic junction)		1	0.3
	Not specified		22	7.5
Deep gray matter №=87 (29.8%)	Nucleocapsular	Basal ganglia	25	8.6
		Internal capsule	12	4.1
		Not specified	7	2.4
	Thalamus		41	14.0
	Hypothalamus		2	0.7
Supratentorial white matter^§^ №=18 (6.2%)	Periventricular	Corpus callosum	6	2.1
		Septum pellucidum	1	0.3
		Corona radiata	3	1.0
		Not specified	3	1.0
	Subcortical	Centrum semiovale	5	1.7
Cerebellar infarcts			4	1.4
Spinal cord infarcts			4	1.4
Cortical cerebral infarcts			18	6.2
Parietal			2	0.7
Temporal			1	0.3
Frontal			1	0.3
Fronto-parieto-occipital			1	0.3
“Middle cerebral artery stroke”			1	0.3
Not specified			12	4.1
Hemorrhage (macroscopic)			32	11.0
Lobar №=21 (7.1%)	Frontal		3	1.0
	Temporal		2	0.7
	Parietal		1	0.3
	Occipital		1	0.3
	Frontoparietal		1	0.3
	Not specified		13	4.5
Deep №=7 (2.4%)	Basal ganglia		6	2.1
	Thalamus		1	0.3
Subarachnoidal			2	0.7
Subdural			1	0.3
Pineal			1	0.3
Other findings			49	16.8
Brain atrophy			16	5.5
Vessel wall enhancement*			≥12*	≥4.1*
PRES-pattern			9	3.1
Optic atrophy			3	1.0
Aneurysms*			≥2*	≥0.7*
Arterial stenosis*			≥2*	≥0.7*
Microhemorrhages*			≥2*	≥0.7*
Leptomeningeal enhancement*			≥1*	≥0.3*
Posterior parietal brain cavernous malformation			1	0.3
Dilatation of the third and lateral ventricles			1	0.3
Ectopic neurohypophysis			1	0.3
Focal cortical anomaly			1	0.3
Total			290	

## Discussion

In this survey of neurological manifestations of DADA2, we found that 50.3% of patients had at least one reported neurological event. The spectrum of neurological manifestations was broad: from ischemic and hemorrhagic strokes to peripheral neuropathy, neuritis, and neurocognitive disorders, such as ASD and learning difficulties.

The mean age of onset of neurological manifestation is 7.2 years, with the youngest patient being only 3 months old and the oldest being 36 years old at the time of neurological manifestations onset. Thus, the onset of neurological manifestations is in line with the onset of DADA2 manifestations in general (7 years), although adult-onset is possible. The late onset can lead to misdiagnosis; therefore, differential diagnosis of DADA2 is obligatory in every case of PAN [48].

Strokes account for 77.5% of all neurological events and, in some cases, can be the initial [2, 13, 21, 23, 24, E6–E14] or even sole [2, 13] manifestation of DADA2. Some neurological signs of CNS involvement can be underestimated due to their mild or transient character (i.e., TIA or “silent” infarcts) or due to the possibility of MRI-negative infarcts, particularly in the brainstem, which may be missed without the use of thin cuts or higher magnetic field strength. Thus, it is crucial to exclude DADA2 whenever a patient presents with an acute stroke at any age, especially in childhood. Besides other non-atherosclerotic arteriopathies, such as arterial dissection, focal cerebral arteriopathy, and Moyamoya vasculopathy, DADA2 should be in the differential diagnosis of arterial ischemic stroke in childhood [E56]. It is also noteworthy that in the past, many patients who would have been diagnosed with Sneddon syndrome are likely to be DADA2 patients.

Interestingly, the preference for the brain stem and deep gray matter structures on MRI is similar to the preferred locations in Behçet’s disease, another immune-mediated vasculitis, albeit that lesions in Behçet’s disease are often not infarcts. This parallel between the imaging pattern of a DADA2-mutation and Behçet’s disease has not been reported on to our knowledge. Yet, an overlap from a clinical perspective [E57] and genetic perspective [E58] has been described before.

Other neurological symptoms and syndromes, such as mononeuropathy and polyneuropathy, and PRES, are also sentinels of DADA2. Mononeuritis multiplex was diagnosed in 12 of the reviewed patients [2, 9, 20, E37, E59, E60]. Caorsi et al. reported that neuropathy of cranial and peripheral nerves was common in DADA2 and that these signs distinguished DADA2 from childhood PAN [10]. A recent paper by Ehlers L. et al. [E61] describes a case of encephalopathy and multifocal neurological deficits in a DADA2 patient previously diagnosed with acute disseminated encephalomyelitis (ADEM). Therefore, it is important to consider the possibility of DADA2 in such patients and maintain a reasonable level of suspicion.

Interestingly, intracranial calcifications, the hallmark of Aicardi-Guttieres syndrome (AGS), the archetypical interferonopathy, are typically absent in DADA2 patients. This is noteworthy considering the involvement of enhanced type I interferon signaling in DADA2 patients and the consideration of SAMHD1 and other AGS underlying genes in the differential diagnosis [16, E62]. The absence of intracranial calcification in DADA2 might suggest that the mechanisms underlying brain abnormalities in interferonopathies may not be applicable to those in DADA2. The other explanation would be that the level of type I IFN upregulation is much higher in typical AGS than in DADA2 [16]. In addition, the exact pathophysiological link between the increased type I IFN and the calcifications in AGS is incompletely understood. It is also important to note that not all interferonopathies are characterized by calcifications.

In addition, patients with DADA2 can have a broad spectrum of subtle neurocognitive disorders, which should be taken into consideration in patients with DADA2, and the evaluation of these conditions should be part of the routine management of DADA2. We hypothesize that the actual number of patients with neurocognitive disorders may be even higher than reported due to underdiagnosis. Our study shows that 22 DADA2 patients (3.5% of all the reviewed patients) had reported neurocognitive disorders. Contrasting the obtained data with the general population, there seems to be a higher prevalence of these disabilities in DADA2. According to a systemic review update by Zeidan J et al., 1% of children have ASD in the general population [E63, E64]. Sayal K et al. state that 5% of all children and adolescents are diagnosed with ADHD. Whether this is only a coincidence, or the discrepancy is due to a direct effect of ADA2 deficiency, or an indirect impact of the context of severe disease is unclear at this stage.

Although neuroimaging findings may show a wide variety, ranging from normal findings to PRES or ADEM-like presentations, the most common manifestation was ischemic and to lesser extent hemorrhagic stroke, presumably reflecting the underlying vasculitis. Especially, lacunar infarcts in the brain stem and deep gray matter were common, in line with previously reported data [1, E22, E32]. It is interesting that the preference for these specific localizations is similar to that of the neurological manifestation of Behçet’s disease, which also has a known clinical [E57] and genetic [E58] overlap with DADA2. Direct signs of underlying vasculitis have also been demonstrated with MRI with vessel wall imaging [E32].

Patients with minor or resolved neurologic symptoms are of particular concern, as half of them do have acute infarcts on MRI (foci of restricted diffusion on MRI) [E65]. They are, therefore, at risk of developing recurrent strokes and disabilities [E66]. The differential diagnosis of ischemic stroke, especially in children, presents a challenge for clinicians, with arteriopathies being the most common cause [80]. As neurological manifestations may lack specificity in DADA2, further investigation is needed to find specific biomarkers, particularly for assessing the risk of stroke, especially in the case of “mild” neurological signs. In the absence of a promising biomarker, the diagnosis of DADA2 requires a high index of suspicion and awareness of the wide phenotypic variability of the disease. We recommend formal exclusion of DADA2 as the underlying condition by both plasma ADA2 enzymatic function testing, as well as ADA2 sequencing [4]. In most patients with absent or severely reduced serum ADA2 enzyme activity, a bi-allelic loss-of-function mutation in the ADA2 gene is found [51].

Early diagnosis offers the opportunity for early initiation of targeted treatment with TNF-α inhibitors which can prevent long-term sequelae due to recurrent ischemic events. In the study of the efficacy of anti-TNF treatment for preventing strokes in patients with DADA2 conducted by Cooray et al., the authors reported the median rate of ischemic events in DADA2 patients to be 2.37 before anti-TNF treatment vs. 0.0 per 100 patient-months after treatment [E5]. Among the biological agents used, etanercept shows the most robust evidence [E31]. Anti-TNF treatment seems to be less effective in reversing the hematological manifestations though [78], E5, E27. Treatment-refractory patients can be cured by hematopoietic stem cell transplantation (HSCT) [81, 93, E67]. At this time, HSCT remains the only available curative option, although gene therapy is on the horizon. In two recent studies [E3, E68], a lentiviral vector-mediated hematopoietic stem and progenitor cells (HSPC) gene therapy has been described to restore ADA2 expression and enzymatic activity in patients’ CD34^+^ cells, normalize secretion of IL-6 and TNFα from macrophages, correcting the inflammatory macrophage phenotype. It is also important to note that aspirin (or other antiplatelet agents) is traditionally not recommended in DADA-2 because of a risk of brain hemorrhage [14, 78].

In conclusion, neurological manifestations affect a significant proportion of patients with DADA2, and the phenotype is broad. Neurological manifestations can be the first and single manifestation of DADA2. Therefore, stroke, encephalitis (including ADEM), PRES, mononeuropathy and polyneuropathy, and Behçet’s disease-like presentations should prompt the neurologist to exclude ADA2 deficiency, especially but not only in childhood. We are convinced that this review will aid in recognizing DADA2 early after the disease onset, which will allow for early treatment initiation and hopefully limit the detrimental outcomes often associated with DADA2.

Additionally, this study has several limitations. The study only included articles written in English, which may have excluded relevant data published in other languages, and therefore could have introduced a language bias into the study. The study only included published articles, which could have resulted in the exclusion of unpublished data or data published in less accessible sources. This could have introduced a publication bias into the study. The quality of the data included in the study could have been influenced by the quality of reporting in the articles reviewed. This could have introduced a reporting bias into the study. The study was a literature review and did not include any primary data collection, which could limit the depth and accuracy of the analysis.

### Supplementary Information

ESM 1 (DOCX 30 kb)

ESM 2 (DOCX 260 kb)

## Data Availability

The datasets generated during and/or analyzed during the current study are available from the corresponding author on reasonable request.
