# Supplementary material for: A Narrative Review of the Neurological Manifestations of Human Adenosine Deaminase 2 Deficiency
Source: J Clin Immunol. 2023 Aug 7;43(8):1916–26. doi: 10.1007/s10875-023-01555-y (PMC10661818; doi:10.1007/s10875-023-01555-y)
Supplement: Supplementary file 1 — (DOCX 30 kb) [file 10875_2023_1555_MOESM1_ESM.docx]

**Supplementary references**

E1. Sharma A, Naidu GSRSNK, Chattopadhyay A, Acharya N, Jha S, Jain S. Novel *CECR1* gene mutations causing deficiency of adenosine deaminase 2, mimicking antiphospholipid syndrome. *Rheumatology*. 2019;58(1):181-182. doi:10.1093/rheumatology/key258

E2. Martin H, Bursztejn AC, Cuny JF, et al. Chronic leg ulcer revealing adenosine deaminase 2 deficiency: an atypical presentation. *Eur J Dermatol EJD*. 2018;28(6):847-848. doi:10.1684/ejd.2018.3427

E3. Zoccolillo M, Brigida I, Barzaghi F, et al. Lentiviral correction of enzymatic activity restrains macrophage inflammation in adenosine deaminase 2 deficiency. *Blood Adv*. 2021;5(16):3174-3187. doi:10.1182/bloodadvances.2020003811

E4. Zhang B, Sun Y, Xu N, et al. Adult-onset deficiency of adenosine deaminase 2-a case report and literature review. *Clin Rheumatol*. 2021;40(10):4325-4339. doi:10.1007/s10067-021-05587-w

E5. Cooray S, Omyinmi E, Hong Y, et al. Anti-tumour necrosis factor treatment for the prevention of ischaemic events in patients with deficiency of adenosine deaminase 2 (DADA2). *Rheumatol Oxf Engl*. 2021;60(9):4373-4378. doi:10.1093/rheumatology/keaa837

E6. Pichard DC, Ombrello AK, Hoffmann P, Stone DL, Cowen EW. Early-onset stroke, polyarteritis nodosa (PAN), and livedo racemosa. *J Am Acad Dermatol*. 2016;75(2):449-453. doi:10.1016/j.jaad.2016.01.057

E7. Ganhão S, Loureiro GB, Oliveira DR, et al. Two cases of ADA2 deficiency presenting as childhood polyarteritis nodosa: novel ADA2 variant, atypical CNS manifestations, and literature review. *Clin Rheumatol*. 2020;39(12):3853-3860. doi:10.1007/s10067-020-05210-4

E8. Trivioli G, Gelain E, Angelotti ML, et al. A Report of 2 Cases of Kidney Involvement in ADA2 Deficiency: Different Disease Phenotypes and the Tissue Response to Type I Interferon. *Am J Kidney Dis*. 2022;80(5):677-682. doi:10.1053/j.ajkd.2022.05.008

E9. Escherich C, Bötticher B, Harmsen S, et al. The Growing Spectrum of DADA2 Manifestations—Diagnostic and Therapeutic Challenges Revisited. *Front Pediatr*. 2022;10:885893. doi:10.3389/fped.2022.885893

E10. Babtiwale S, Vishnu V, Garg A, Goyal V, Singh M, Padma Srivastava M. Young stroke and systemic manifestations: Deficiency of adenosine deaminase-2 (DADA-2). *Ann Indian Acad Neurol*. 2021;24(6):977. doi:10.4103/aian.AIAN_657_20

E11. Jha A, Doley D, Mathew J, Hegde AG. Deficiency of adenosine deaminase 2 (DADA2): two cases of multisystem vasculitis managed in a South Indian tertiary care centre. *BMJ Case Rep*. 2021;14(8):e242963. doi:10.1136/bcr-2021-242963

E12. Vai S, Marin E, Cosso R, et al. A Novel Germline Mutation of ADA2 Gene in Two “Discordant” Homozygous Female Twins Affected by Adenosine Deaminase 2 Deficiency: Description of the Bone-Related Phenotype. *Int J Mol Sci*. 2021;22(15):8331. doi:10.3390/ijms22158331

E13. Price-Kuehne F, Close R, Armon K. Early-onset stroke and iron-deficiency anaemia in young siblings. *Rheumatology*. 2021;60(Supplement_3):iii15-iii17. doi:10.1093/rheumatology/keab058

E14. Ayan G, Yagiz B, Cinar O, et al. A novel variant in severe disease of DADA2: involving vasculitic and haematologic features. *Scand J Rheumatol*. 2023;52(1):93-95. doi:10.1080/03009742.2022.2095724

E15. Van Montfrans JM, Hartman EAR, Braun KPJ, et al. Phenotypic variability in patients with ADA2 deficiency due to identical homozygous R169Q mutations. *Rheumatology*. 2016;55(5):902-910. doi:10.1093/rheumatology/kev439

E16. Fellmann F, Angelini F, Wassenberg J, et al. IL-17 receptor A and adenosine deaminase 2 deficiency in siblings with recurrent infections and chronic inflammation. *J Allergy Clin Immunol*. 2016;137(4):1189-1196.e2. doi:10.1016/j.jaci.2015.07.053

E17. Sahin S, Adrovic A, Barut K, et al. Clinical, imaging and genotypical features of three deceased and five surviving cases with ADA2 deficiency. *Rheumatol Int*. 2018;38(1):129-136. doi:10.1007/s00296-017-3740-3

E18. Sozeri B, Ercan G, Dogan OA, Yıldız J, Demir F, Doğanay L. The same mutation in a family with adenosine deaminase 2 deficiency. *Rheumatol Int*. 2021;41(1):227-233. doi:10.1007/s00296-019-04444-z

E19. Pardinhas C, Santo G, Escada L, et al. A Case of Deficiency of Adenosine Deaminase 2: 28 years of Diagnostic Challenges. *Case Rep Nephrol Dial*. 2021;11(3):340-347. doi:10.1159/000517141

E20. Patra PK, Mondal SK, Singhal M, et al. Deficiency of Adenosine Deaminase 2—a Monogenic Cause of Wunderlich Syndrome. *J Clin Immunol*. 2021;41(7):1693-1695. doi:10.1007/s10875-021-01097-1

E21. Akgun‐Dogan O, Simsek‐Kiper PO, Taskiran E, et al. ADA2 deficiency in a patient with Noonan syndrome‐like disorder with loose anagen hair: The co‐occurrence of two rare syndromes. *Am J Med Genet A*. 2019;179(12):2474-2480. doi:10.1002/ajmg.a.61363

E22. Bulut K, Erden E, Karadag A, Oguz K, Ozen S. Deficiency of ADA2: special focus on CNS imaging. Published online 2018:1169 words. doi:10.1594/ECR2018/C-1833

E23. Uettwiller F, Sarrabay G, Rodero MP, et al. ADA2 deficiency: case report of a new phenotype and novel mutation in two sisters. *RMD Open*. 2016;2(1):e000236. doi:10.1136/rmdopen-2015-000236

E24. Skrabl-Baumgartner A, Plecko B, Schmidt WM, et al. Autoimmune phenotype with type I interferon signature in two brothers with ADA2 deficiency carrying a novel CECR1 mutation. *Pediatr Rheumatol*. 2017;15(1):67. doi:10.1186/s12969-017-0193-x

E25. Poswar F de O, da Fonseca RMT, de Albuquerque LCP, et al. Adenosine deaminase 2 deficiency presenting as spastic paraplegia and systemic vasculitis. *J Neurol*. 2016;263(4):818-820. doi:10.1007/s00415-016-8070-y

E26. Kisla Ekinci RM, Anlas O, Ozalp O. Clinical presentation of children with Deficiency of Adenosine deaminase 2: A case series. *Eur J Med Genet*. 2022;65(8):104555. doi:10.1016/j.ejmg.2022.104555

E27. Lee PY, Kellner ES, Huang Y, et al. Genotype and functional correlates of disease phenotype in deficiency of adenosine deaminase 2 (DADA2). *J Allergy Clin Immunol*. 2020;145(6):1664-1672.e10. doi:10.1016/j.jaci.2019.12.908

E28. Veryser E, Meyts I, Casteels I, Demaerel P, De Somer L, Cassiman C. Two Cases Presenting With Unilateral Adduction Deficit Associated With Human Adenosine Deaminase 2 Deficiency. *J Pediatr Ophthalmol Strabismus*. 2021;58(4). doi:10.3928/01913913-20210416-02

E29. Al-Hebshi A, Aljohani M, AlShenaifi N, Aloqbi M, Turkistani W, Hakami F. A Novel Variant of Adenosine Deaminase 2 Deficiency Presented With Chronic Thrombocytopenia, Anemia, and Early-Onset Stroke. *Cureus*. Published online May 28, 2021. doi:10.7759/cureus.15288

E30. Guo L, Wang J, Yang X, et al. Novel ADA2 Compound Heterozygous Mutations Resulting in Deficiency of Adenosine Deaminase 2 in a Pair of Siblings. *J Clin Immunol*. 2021;41(4):837-842. doi:10.1007/s10875-021-00981-0

E31. Ombrello AK, Qin J, Hoffmann PM, et al. Treatment Strategies for Deficiency of Adenosine Deaminase 2. *N Engl J Med*. 2019;380(16):1582-1584. doi:10.1056/NEJMc1801927

E32. Geraldo AF, Caorsi R, Tortora D, et al. Widening the Neuroimaging Features of Adenosine Deaminase 2 Deficiency. *Am J Neuroradiol*. 2021;42(5):975-979. doi:10.3174/ajnr.A7019

E33. Betrains A, Staels F, Moens L, et al. Diagnosis of deficiency of adenosine deaminase type 2 in adulthood. *Scand J Rheumatol*. 2021;50(6):493-496. doi:10.1080/03009742.2021.1881156

E34. Krutzke S, Horneff G. Treatment of Two Boys Suffering From Deficiency of Adenosine Deaminase Type 2 (DADA2) With TNF-Inhibitor Etanercept. *JCR J Clin Rheumatol*. 2021;27(8S):S509-S512. doi:10.1097/RHU.0000000000001145

E35. Lamprecht P, Humrich JY, Diebold I, Riemekasten G. Diagnosis of deficiency of adenosine deaminase 2 with early onset polyarteritis nodosa in an adult patient with a novel compound heterozygous CECR1 mutation. *Clin Exp Rheumatol*. 2018;36 Suppl 111(2):177.

E36. Tull TJ, Martin B, Spencer J, et al. Sneddon syndrome associated with two novel ADA2 gene mutations. *Rheumatology*. 2020;59(6):1448-1450. doi:10.1093/rheumatology/kez446

E37. Gibson KM, Morishita KA, Dancey P, et al. Identification of Novel Adenosine Deaminase 2 Gene Variants and Varied Clinical Phenotype in Pediatric Vasculitis. *Arthritis Rheumatol*. 2019;71(10):1747-1755. doi:10.1002/art.40913

E38. Wang W, Zhang T, Zheng W, et al. Diagnosis and management of adenosine deaminase 2 deficiency children: the experience from China. *Pediatr Rheumatol*. 2021;19(1):44. doi:10.1186/s12969-021-00535-z

E39. Bras J, Guerreiro R, Santo GC. Mutant ADA2 in Vasculopathies. *N Engl J Med*. 2014;371(5):478-481. doi:10.1056/NEJMc1405506

E40. Nanthapisal S, Murphy C, Omoyinmi E, et al. Deficiency of Adenosine Deaminase Type 2: A Description of Phenotype and Genotype in Fifteen Cases: ADA2 deficiency. *Arthritis Rheumatol*. 2016;68(9):2314-2322. doi:10.1002/art.39699

E41. Chong-Neto HJ, Segundo GRS, Bandeira M, et al. Homozygous Splice ADA2 Gene Mutation Causing ADA-2 Deficiency. *J Clin Immunol*. 2019;39(8):842-845. doi:10.1007/s10875-019-00697-2

E42. Schnappauf O, Zhou Q, Moura NS, et al. Deficiency of Adenosine Deaminase 2 (DADA2): Hidden Variants, Reduced Penetrance, and Unusual Inheritance. *J Clin Immunol*. 2020;40(6):917-926. doi:10.1007/s10875-020-00817-3

E43. Liu L, Wang W, Wang Y, et al. A Chinese DADA2 patient: report of two novel mutations and successful HSCT. *Immunogenetics*. 2019;71(4):299-305. doi:10.1007/s00251-018-01101-w

E44. Çakan M, Aktay-Ayaz N, Karadağ ŞG, et al. Atypical phenotype of an old disease or typical phenotype of a new disease: deficiency of adenosine deaminase 2. *Turk J Pediatr*. 2019;61(3):413. doi:10.24953/turkjped.2019.03.014

E45. Barzaghi F, Minniti F, Mauro M, et al. ALPS-Like Phenotype Caused by ADA2 Deficiency Rescued by Allogeneic Hematopoietic Stem Cell Transplantation. *Front Immunol*. 2019;9:2767. doi:10.3389/fimmu.2018.02767

E46. Van Eyck L, Hershfield MS, Pombal D, et al. Hematopoietic stem cell transplantation rescues the immunologic phenotype and prevents vasculopathy in patients with adenosine deaminase 2 deficiency. *J Allergy Clin Immunol*. 2015;135(1):283-287.e5. doi:10.1016/j.jaci.2014.10.010

E47. Süleyman M, Tan Ç, Uner A, et al. Adenosine Deaminase Type II Deficiency: Severe Chronic Neutropenia, Lymphoid Infiltration in Bone Marrow, and Inflammatory Features. *Immunol Invest*. 2022;51(3):558-566. doi:10.1080/08820139.2020.1853153

E48. Le Voyer T, Boutboul D, Ledoux-Pilon A, et al. Late-Onset EBV Susceptibility and Refractory Pure Red Cell Aplasia Revealing DADA2. *J Clin Immunol*. 2020;40(6):948-953. doi:10.1007/s10875-020-00812-8

E49. Pinto e Vairo F, Koster MJ, Kemppainen JL, Thomas BC, Warrington KJ. Comment on: Anti-tumour necrosis factor treatment for the prevention of ischaemic events in patients with deficiency of adenosine deaminase 2 (DADA2). *Rheumatology*. 2021;60(6):e218-e219. doi:10.1093/rheumatology/keab081

E50. Gonçalves T da S, Alves CAPF, da Paz JA, Lucato LT. Teaching NeuroImages: Lacunar stroke and polyarteritis nodosa: Consider ADA2 deficiency (DADA2). *Neurology*. 2019;92(15):e1801-e1802. doi:10.1212/WNL.0000000000007270

E51. Tekgöz N, Aydın F, Kurt T, et al. Clinical features and outcomes of childhood polyarteritis nodosa: A single referral center experience. *Mod Rheumatol*. 2021;31(6):1142-1147. doi:10.1080/14397595.2021.1886892

E52. Schepp J, Bulashevska A, Mannhardt-Laakmann W, et al. Deficiency of Adenosine Deaminase 2 Causes Antibody Deficiency. *J Clin Immunol*. 2016;36(3):179-186. doi:10.1007/s10875-016-0245-x

E53. Lee PY, Huang Y, Zhou Q, et al. Disrupted N-linked glycosylation as a disease mechanism in deficiency of ADA2. *J Allergy Clin Immunol*. 2018;142(4):1363-1365.e8. doi:10.1016/j.jaci.2018.05.038

E54. Garg N, Kasapcopur O, Foster J, et al. Novel adenosine deaminase 2 mutations in a child with a fatal vasculopathy. *Eur J Pediatr*. 2014;173(6):827-830. doi:10.1007/s00431-014-2320-8

E55. Carneiro DR, Rebelo O, Matos A, et al. Vasculitic peripheral neuropathy in deficiency of adenosine deaminase 2. *Neuromuscul Disord*. 2021;31(9):891-895. doi:10.1016/j.nmd.2021.05.001

E56. Sporns PB, Fullerton HJ, Lee S, et al. Childhood stroke. *Nat Rev Dis Primer*. 2022;8(1):12. doi:10.1038/s41572-022-00337-x

E57. van Well GTJ, Kant B, van Nistelrooij A, et al. Phenotypic variability including Behçet’s disease-like manifestations in DADA2 patients due to a homozygous c.973-2A>G splice site mutation. *Clin Exp Rheumatol*. 2019;37 Suppl 121(6):142-146.

E58. Burillo-Sanz S, Montes-Cano MA, García-Lozano JR, et al. Mutational profile of rare variants in inflammasome-related genes in Behçet disease: A Next Generation Sequencing approach. *Sci Rep*. 2017;7(1):8453. doi:10.1038/s41598-017-09164-7

E59. Liebowitz J, Hellmann DB, Schnappauf O. Thirty Years of Followup in 3 Patients with Familial Polyarteritis Nodosa due to Adenosine Deaminase 2 Deficiency. *J Rheumatol*. 2019;46(8):1059-1060. doi:10.3899/jrheum.180820

E60. Pimpale Chavan P, Ramadoss D, Khan A, Lee PY, Khubchandani R. Deficiency of Adenosine Deaminase 2 (DADA2): One Disease, Several Faces. *Indian J Pediatr*. 2021;88(8):828-830. doi:10.1007/s12098-021-03809-2

E61. Ehlers L, Bucciol G, KU Leuven - UZA DADA2 team, et al. ADA2 Deficiency Mimicking Acute Disseminated Encephalomyelitis. *J Clin Immunol*. Published online December 6, 2022. doi:10.1007/s10875-022-01413-3

E62. Zeidan J, Fombonne E, Scorah J, et al. Global prevalence of autism: A systematic review update. *Autism Res Off J Int Soc Autism Res*. 2022;15(5):778-790. doi:10.1002/aur.2696

E63. Sayal K, Prasad V, Daley D, Ford T, Coghill D. ADHD in children and young people: prevalence, care pathways, and service provision. *Lancet Psychiatry*. 2018;5(2):175-186. doi:10.1016/S2215-0366(17)30167-0

E64. Coutts SB, Hill MD, Simon JE, Sohn CH, Scott JN, Demchuk AM. Silent ischemia in minor stroke and TIA patients identified on MR imaging. *Neurology*. 2005;65(4):513-517. doi:10.1212/01.WNL.0000169031.39264.ff

E65. Asdaghi N, Campbell BCV, Butcher KS, et al. DWI Reversal Is Associated with Small Infarct Volume in Patients with TIA and Minor Stroke. *Am J Neuroradiol*. 2014;35(4):660-666. doi:10.3174/ajnr.A3733

E66. Hashem H, Bucciol G, Ozen S, et al. Hematopoietic Cell Transplantation Cures Adenosine Deaminase 2 Deficiency: Report on 30 Patients. *J Clin Immunol*. 2021;41(7):1633-1647. doi:10.1007/s10875-021-01098-0

E67. Hong Y, Casimir M, Houghton BC, et al. Lentiviral Mediated ADA2 Gene Transfer Corrects the Defects Associated With Deficiency of Adenosine Deaminase Type 2. *Front Immunol*. 2022;13:852830. doi:10.3389/fimmu.2022.852830

E68. Van Eyck L, Liston A, Wouters C. Mutant ADA2 in Vasculopathies. *N Engl J Med*. 2014 Jul 31;371(5):480. doi:10.1056/NEJMc1405506

E69. Garg N, Kasapcopur O, Foster J, Barut K, Tekin A, Kızılkılıç O, et al. Novel adenosine deaminase 2 mutations in a child with a fatal vasculopathy. *Eur J Pediatr*. 2014 Jun;173(6):827–30. doi:10.1007/s00431-014-2320-8

E70. Batu ED, Karadag O, Taskiran EZ, Kalyoncu U, Aksentijevich I, Alikasifoglu M, et al. A Case Series of Adenosine Deaminase 2-deficient Patients Emphasizing Treatment and Genotype-phenotype Correlations. *J Rheumatol*. 2015 Aug;42(8):1532–4. doi:10.3899/jrheum.150024

E71. Sahin S, Adrovic A, Barut K, Ugurlu S, Turanli ET, Ozdogan H, et al. Clinical, imaging and genotypical features of three deceased and five surviving cases with ADA2 deficiency. *Rheumatol Int*. 2018 Jan;38(1):129–36. doi:10.1007/s00296-017-3740-3

E72. Tanatar A, Karadağ ŞG, Sözeri B, Sönmez HE, Çakan M, Kendir Demirkol Y, et al. ADA2 Deficiency: Case Series of Five Patients with Varying Phenotypes. *J Clin Immunol*. 2020 Feb;40(2):253–8. doi:10.1007/s10875-019-00734-0

E73. Sahin S, Adrovic A, Barut K, Baran S, Tahir Turanli E, Canpolat N, et al. A 9.5-year-old boy with recurrent neurological manifestations and severe hypertension, treated initially for polyarteritis nodosa, was subsequently diagnosed with adenosine deaminase type 2 deficiency (DADA2) which responded to anti-TNF-α. *Paediatr Int Child Health*. 2020 Jan 2;40(1):65–8. doi:10.1080/20469047.2018.1559495

E74. Kisla Ekinci RM, Balci S, Hershfield M, Bisgin A, Dogruel D, Altintas DU, Yilmaz M. Deficiency of adenosine deaminase 2: a case series revealing clinical manifestations, genotypes and treatment outcomes from Turkey. *Rheumatology (Oxford)*. 2020 Jan 1;59(1):254-256. doi:10.1093/rheumatology/kez260

E75. Van Nieuwenhove E, Humblet-Baron S, Van Eyck L, De Somer L, Dooley J, Tousseyn T, Hershfield M, Liston A, Wouters C. ADA2 Deficiency Mimicking Idiopathic Multicentric Castleman Disease. *Pediatrics*. 2018 Sep;142(3):e20172266. doi:10.1542/peds.2017-2266

E76. Kurumoğlu İncekalan T, Kışla Ekinci RM, Naz Şimdivar GH, Doğan NÇ, Çiloğlu E. Evaluation of subclinical ocular involvement in patients with deficiency of adenosine deaminase 2 (DADA2). *Clin Rheumatol*. 2022 Aug;41(8):2533-2540. doi:10.1007/s10067-022-06194-z

E77. Iyengar VV, Chougule A, Gowri V, Taur P, Prabhu S, Bodhanwala M, Desai MM. DADA2 presenting as nonimmune hemolytic anemia with recurrent macrophage activation syndrome. *Pediatr Blood Cancer*. 2022 Jun;69(6):e29461. doi:10.1002/pbc.29461

E78. Van Eyck L, Liston A, Meyts I. Mutant ADA2 in Vasculopathies. *N Engl J Med*. 2014 Jul;371(5):478-479. doi:10.1056/nejmc1405506

E79. Van Montfrans JM, Zavialov A, Zhou Q. Mutant ADA2 in Vasculopathies. *N Engl J Med*. 2014 Jul 31;371(5):478. doi:10.1056/nejmc1405506

E80. Hashem H, Kumar AR, Müller I, Babor F, Bredius R, Dalal J, et al. Deficiency of Adenosine Deaminase Type 2 Foundation. Hematopoietic stem cell transplantation rescues the hematological, immunological, and vascular phenotype in DADA2. *Blood*. 2017 Dec 14;130(24):2682-2688. doi:10.1182/blood-2017-07-798660

E81. Ghurye RR, Sundaram K, Smith F, Clark B, Simpson MA, Fairbanks L, et al. Novel ADA2 mutation presenting with neutropenia, lymphopenia and bone marrow failure in patients with deficiency in adenosine deaminase 2 (DADA2). *Br J Haematol*. 2019 Aug;186(3):e60-e64. doi:10.1111/bjh.15896

E82. Maccora I, Frongia I, Azzari C, Ricci S, Cimaz R, Simonini G. A misleading case of deficiency of adenosine deaminase 2 (DADA2): the magnifying glass of the scientific knowledge drives the tailored medicine in real life. *Clin Exp Rheumatol*. 2018;36(6 Suppl 115):146.

E83. Staples E, Simeoni I, Stephens JC, Allen HL, Wright P, Davies EG, et al. ADA2 deficiency complicated by EBV-driven lymphoproliferative disease. *J Clin Immunol*. 2020 Jun;215:108443. doi:10.1016/j.clim.2020.108443

E84. Arts K, Bergerson JRE, Ombrello AK, Similuk M, Oler AJ, Agharahimi A, et al. Warts and DADA2: a Mere Coincidence? *J Clin Immunol*. 2018 Nov;38(8):836-843. doi:10.1007/s10875-018-0565-0

E85. Deuitch NT, Yang D, Lee PY, Yu X, Moura NS, Schnappauf O, et al. TNF inhibition in vasculitis management in adenosine deaminase 2 deficiency (DADA2). *J Allergy Clin Immunol*. 2022 May;149(5):1812-1816.e6. doi:10.1016/j.jaci.2021.10.030

E86. Yap JY, Moens L, Lin MW, Kane A, Kelleher A, Toong C, et al. Intrinsic Defects in B Cell Development and Differentiation, T Cell Exhaustion and Altered Unconventional T Cell Generation Characterize Human Adenosine Deaminase Type 2 Deficiency. *J Clin Immunol*. 2021 Nov;41(8):1915–35. doi:10.1007/s10875-021-01141-0

E87. Watanabe N, Gao S, Wu Z, Batchu S, Kajigaya S, Diamond C, et al. Analysis of deficiency of adenosine deaminase 2 pathogenesis based on single‐cell RNA sequencing of monocytes. *J Leukoc Biol*. 2021 Sep;110(3):409–24. doi:10.1002/JLB.3HI0220-119RR

E88. Sundin M, Marits P, Nierkens S, Kolios AGA, Nilsson J. “Immune” Thrombocytopenia as Key Feature of a Novel ADA2 Deficiency Variant: Implication on Differential Diagnostics of ITP in Children. *J Pediatr Hematol Oncol*. 2019 Mar;41(2):155–7. doi:10.1097/MPH.0000000000001132

E89. Abbasi A, Batllori M, Gil-Sáez FJ, Rodríguez-Pintó I, Antón López J, Iglesias Jímenez E. Importancia de la determinación de actividad enzimática en el diagnóstico del déficit de adenosina desaminasa 2 (DADA2). *Med Clínica*. 2022 Sep;159(6):283–6. doi:10.1016/j.medcli.2021.12.020

E90. Günthner R, Wagner M, Thurm T, Ponsel S, Höfele J, Lange-Sperandio B. Identification of co-occurrence in a patient with Dent’s disease and ADA2-deficiency by exome sequencing. *Gene*. 2018 Apr;649:23–6. doi:10.1016/j.gene.2018.01.060

E91. Dimachkie MD, Fraga GR, Moura NS, Springer JM. A Rare Case of Adenosine Deaminase 2 Deficiency Presenting With Temporal Arteritis. *JCR J Clin Rheumatol*. 2021 Oct;27(7):e251–2. doi:10.1097/RHU.0000000000001384

E92. Trentin F, Carli L, Tani C, Mosca M. Unveiling Deficiency of Adenosine Deaminase 2: An Adult Patient With Recurrent Strokes, Vasculitic Ulcers, and Bowel Perforation. *J Rheumatol*. 2022 Jul 1;jrheum.220218. doi:10.3899/jrheum.220218

E93. Springer JM, Gierer SA, Jiang H, Kleiner D, Deuitch N, Ombrello AK, et al. Deficiency of Adenosine Deaminase 2 in Adult Siblings: Many Years of a Misdiagnosed Disease With Severe Consequences. *Front Immunol*. 2018 Jun 14;9:1361. doi:10.3389/fimmu.2018.01361

E94. Zhao X, Zhang J, Li C, Kuang W, Deng J, Tan X, Li C, Li S, Wang J. Early onset is an indication of the severity of DADA2 disease*. Rheumatology (Oxford).* 2023 Feb 1;62(2):969-976. doi:10.1093/rheumatology/keac233.

E95. Hashem H, Egler R, Dalal J. Refractory Pure Red Cell Aplasia Manifesting as Deficiency of Adenosine Deaminase 2. *J Pediatr Hematol Oncol*. 2017 Jul;39(5):e293-e296. doi:10.1097/MPH.0000000000000805.

E96. Insalaco A, Moneta GM, Pardeo M, Caiello I, Messia V, Bracaglia C, et al. Variable Clinical Phenotypes and Relation of Interferon Signature with Disease Activity in ADA2 Deficiency. J Rheumatol. 2019 May;46(5):523–6. doi: 10.3899/jrheum.180045

E97. Ben-Ami T, Revel-Vilk S, Brooks R, Shaag A, Hershfield MS, Kelly SJ, et al. Extending the Clinical Phenotype of Adenosine Deaminase 2 Deficiency. *J Pediatr*. 2016 Oct;177:316–20. doi:10.1016/j.jpeds.2016.06.058

E98. Khubchandani R, Aksentijevich I. Deficiency of Adenosine Deaminase 2 (DADA2) - A New Autoinflammatory Disease with Multisystem Features. *Indian Pediatr*. 2020 Nov 15;57(11):1074–5.

E99. Göschl L, Winkler S, Dmytrus J, Heredia RJ, Lagler H, Ramharter M, et al. Unreported Missense Mutation in the Dimerization Domain of ADA2 Leads to ADA2 Deficiency Associated with Severe Oral Ulcers and Neutropenia in a Female Somalian Patient—Addendum to the Genotype-Phenotype Puzzle. *J Clin Immunol*. 2020 Jan;40(1):223–6. doi:10.1007/s10875-019-00700-w

E100. Alaygut D. A Child Diagnosed With Treatment-Resistant Polyarteritis Nodosa: Can the Clinical Diagnosis Be Different? *Arch Rheumatol*. 2019 Sep 2;34(3):338–42. doi:10.5606/ArchRheumatol.2019.7075

E101. Alabbas F, Elyamany G, Alsharif O, Hershfield M, Meyts I. Childhood Hodgkin Lymphoma: Think DADA2. *J Clin Immunol*. 2019 Jan;39(1):26–9. doi:10.1007/s10875-019-0590-7

E102. Albalawi R, Hanafy E, Alnafea H, et al. Novel Adenosine Deaminase 2 (ADA2) Mutations Associated With Hematological Manifestations. *J Investig Med High Impact Case Rep*. 2021;9:23247096211056770. doi:10.1177/23247096211056770

E103. Al-Shaikh R, Alnowaiser D, Peer-Zada AA, Almutairi A, Alghamdi H. Atypical presentation of adenosine deaminase 2 deficiency with bi-allelic ADA2 mutation [published correction appears in Clin Case Rep. 2022 Jul 14;10(7):e6068]. *Clin Case Rep*. 2022;10(3):e05408. Published 2022 Mar 1. doi:10.1002/ccr3.5408

E104. Alsultan A, Basher E, Alqanatish J, Mohammed R, Alfadhel M. Deficiency of ADA2 mimicking autoimmune lymphoproliferative syndrome in the absence of livedo reticularis and vasculitis. *Pediatr Blood Cancer*. 2018;65(4):10.1002/pbc.26912. doi:10.1002/pbc.26912

E105. Çakan M, Sözeri B. Deficiency of Adenosine Deaminase 2 Presenting as Periodic Fever at the Mainland of Familial Mediterranean Fever. *J Clin Immunol*. 2022;42(7):1409-1410. doi:10.1007/s10875-022-01328-z

E106. Keer N, Hershfield M, Caskey T, Unizony S. Novel compound heterozygous variants in CECR1 gene associated with childhood onset polyarteritis nodosa and deficiency of ADA2. *Rheumatology (Oxford)*. 2016;55(6):1145-7. doi:10.1093/rheumatology/kew050

E107. Cipe FE, Aydogmus C, Serwas NK, Keskindemirci G, Boztuğ K. Novel Mutation in CECR1 Leads to Deficiency of ADA2 with Associated Neutropenia. *J Clin Immunol*. 2018;38(3):273-7. doi:10.1007/s10875-018-0487-x

E108. Ekinci RMK, Balcı S, Bisgin A, et al. A homozygote novel L451W mutation in CECR1 gene causes deficiency of adenosine deaminase 2 in a pediatric patient representing with chronic lymphoproliferation and cytopenia. *Pediatr Hematol Oncol*. 2019;36(6):376-381. doi:10.1080/08880018.2019.1621973

E109. Gonzalez Santiago TM, Zavialov A, Saarela J, et al. Dermatologic Features of ADA2 Deficiency in Cutaneous Polyarteritis Nodosa [published correction appears in JAMA Dermatol. 2016 Sep 1;152(9):1065] [published correction appears in JAMA Dermatol. 2016 Sep 1;152(9):1065]. *JAMA Dermatol*. 2015;151(11):1230-1234. doi:10.1001/jamadermatol.2015.1635

E110. Claassen D, Boals M, Bowling KM, et al. Complexities of genetic diagnosis illustrated by an atypical case of congenital hypoplastic anemia. *Cold Spring Harb Mol Case Stud*. 2018;4(6):a003384. Published 2018 Dec 17. doi:10.1101/mcs.a003384

E111. Hsu AP, West RR, Calvo KR, et al. Adenosine deaminase type 2 deficiency masquerading as GATA2 deficiency: Successful hematopoietic stem cell transplantation. *J Allergy Clin Immunol*. 2016;138(2):628-630.e2. doi:10.1016/j.jaci.2016.03.016

E112. Kisla Ekinci RM, Balci S, Bisgin A, et al. Renal Amyloidosis in Deficiency of Adenosine Deaminase 2: Successful Experience With Canakinumab. *Pediatrics*. 2018;142(5):e20180948. doi:10.1542/peds.2018-0948

E113. Grossi A, Cusano R, Rusmini M, et al. ADA2 deficiency due to a novel structural variation in 22q11.1. *Clin Genet*. 2019;95(6):732-733. doi:10.1111/cge.13518

E114. Keino D, Kondoh K, Kim Y, et al. Successful treatment with cyclosporine and anti-tumour necrosis factor agent for deficiency of adenosine deaminase-2. *Scand J Rheumatol*. 2021;50(3):243-245. doi:10.1080/03009742.2020.1772868

E115. Brooks JP, Rice AJ, Ji W, et al. Uncontrolled Epstein-Barr Virus as an Atypical Presentation of Deficiency in ADA2 (DADA2). *J Clin Immunol.* 2021;41(3):680-683. doi:10.1007/s10875-020-00940-1

E116. Nihira H, Nakagawa K, Izawa K, et al. Fever of unknown origin with rashes in early infancy is indicative of adenosine deaminase type 2 deficiency. *Scand J Rheumatol.* 2018;47(2):170-172. doi:10.1080/03009742.2017.1324912

E117. Saettini F, Fazio G, Corti P, et al. Two siblings presenting with novel ADA2 variants, lymphoproliferation, persistence of large granular lymphocytes, and T-cell perturbations. *Clin Immunol*. 2020;218:108525. doi:10.1016/j.clim.2020.108525

E118. Ferriani MPL, Valera ET, de Sousa GR, et al. ADA2 deficiency (DADA2) associated with Evans syndrome and a severe ADA2 genotype. *Rheumatology (Oxford).* 2021;60(7):e237-e239. doi:10.1093/rheumatology/keab011

E119. Dell'Orso G, Grossi A, Penco F, et al. Case Report: Deficiency of Adenosine Deaminase 2 Presenting With Overlapping Features of Autoimmune Lymphoproliferative Syndrome and Bone Marrow Failure. *Front Immunol*. 2021;12:754029. Published 2021 Oct 14. doi:10.3389/fimmu.2021.754029
