# Supplementary material for: A Narrative Review of the Neurological Manifestations of Human Adenosine Deaminase 2 Deficiency
Source: J Clin Immunol. 2023 Aug 7;43(8):1916–26. doi: 10.1007/s10875-023-01555-y (PMC10661818; doi:10.1007/s10875-023-01555-y)
Supplement: Supplementary file 2 — (DOCX 260 kb) [file 10875_2023_1555_MOESM2_ESM.docx]

**Appendix**

**Table S1. Genetic variants identified in 495 patients with DADA2**

| cDNA | pNomen | № of patients with homozygous mutation | № of patients with heterozygous mutation | Type | References |
| --- | --- | --- | --- | --- | --- |
| c.139G>A | p.(Gly47Arg) | 78 | 24 | Missense | [1,2,10,16,20,99,E3,E5,E18,E24-E26,E31,E32,E34,E37,E44,E60,E67-E77] |
| c.506G>A | p.(Arg169Gln) | 34 | 52 | Missense | [1,2,8,9,16,21,23,97,99,E5,E9,E13,E15,E28, E31,E33,E38,E40,E46,E49,E52,E60,E66,E78-E88] |
|  | p.(Gly47Arg) | 21 | 4 | Missense | [9,22,E10,E11,E51,E85,E86,E89] |
| c.752C>T | p.(Pro251Leu) | 10 | 14 | Missense | [2,10,13,E5,E40,E67,E89-E91] |
| c.1078A>G | p.(Thr360Ala) | 10 | 15 | Missense | [10,E26,E31,E32,E39,E55,E85,E87,E92] |
| c.973-2A>G | p.? | 9 | 11 | Splice site | [99,97,8,E84,E86,E33,E93,E7,E57,E41] |
| c.1072G>A | p.(Gly358Arg) | 9 | 10 | Missense | [9,13,20,E27,E31,E38,E60,E66,E80,E85, E94,E95] |
| c.1358A>G | p.(Tyr453Cys) | 9 | 23 | Missense | [1,10,99,E2,E5,E9,E19, E23,E25,E31,E34,E38,E42,E74,E86,E87,E96] |
| c.143dup | p.(Arg49Alafs*13) | 6 | 2 | Frameshift | [20,E27,E77,E97] |
| c.139G>C | p.(Gly47Arg) | 6 | 2 | Missense | [18,E20,E37,E40,E98] |
| c.139G>T | p.(Gly47Trp) | 5 | 2 | Missense | [E5,E27,E35,E66,E67,E99] |
| c.505C>G | p.(Arg169Gly) | 4 | 7 | Missense | [E5,E30,E52,E94] |
| c.140G>C | p.(Gly47Ala) | 4 | 11 | Missense | [1,10,23,97,E6,E31,E37,E87,E90,E100] |
| c.533T>C | p.(Phe178Ser) | 4 | 0 | Missense | [E27,E97] |
| c.1447_1451del | p.(Ser483Profs*5) | 4 | 0 | Frameshift | [E29,E101,E102] |
|  | p.(Thr317Argfs*25) | 4 | 0 | Frameshift | [E74,E76] |
| c.1397_1403delAGGCTGA | p.(Lys466Thrfs*2) | 3 | 0 | Frameshift | [E27,E47,E86] |
| c.1373T>A | p.(Val458Asp) | 3 | 4 | Missense | [97,E7,E72,E93] |
| c.882-2A>G | p.? | 2 | 0 | Splice site | [E103,E104] |
| c.232_322+105delinsATG | p.? | 2 | 0 | Deletion-insertion | [E5] |
| c.144del | p.(Arg49Glyfs*4) | 2 | 8 | Frameshift | [E3,E5,E8,E40,E80,E91,E105] |
| c.982G>A | p.(Glu328Lys) | 2 | 1 | Missense | [E51,E106] |
| c.73G>T | p.(Gly25Cys) | 2 | 0 | Missense | [99] |
| c.962G>A | p.(Gly321Glu) | 2 | 0 | Missense | [E27,E107] |
| c.140G>T | p.(Gly47Val) | 2 | 14 | Missense | [2,10,20,E3,E11,E28,E32,E33,E66,E80,E86] |
| c.1052T>A | p.(Leu351Gln) | 2 | 0 | Missense | [E37] |
| c.1352T>G | p.(Leu451Trp) | 2 | 0 | Missense | [E76,E108] |
| c.1392dup | p.(Met465Aspfs*4) | 2 | 0 | Frameshift | [20,E47] |
|  | p.(Tyr227Cys) | 2 | 0 | Missense | [E5,E67] |
| c.1367A>G | p.(Tyr456Cys) | 2 | 2 | Missense | [20,E3,E35,E45] |
| Unspecified deletion |  | 2 | 7 | Deletion | [1,E16,E31,E37] |
| exon 7 deletion |  | 2 | 5 | Deletion | [96,E4,E38,E59,E66] |
| c.973-1G>A | p.? | 1 | 0 | Splice site | [97] |
| c.714_738dup | p.(Ala247Glnfs*16) | 1 | 0 | Frameshift | [E102] |
| c.1069G>A | p.(Ala357Thr) | 1 | 2 | Missense | [E31,E37,E87] |
| c.393delG | p.(Arg131Serfs*52) | 1 | 1 | Frameshift | [E38,E66] |
| c.916C>T | p.(Arg306*) | 1 | 3 | Nonsense | [20,E9,E69,E71] |
| c.1110C>A | p.(Asn370Lys) | 1 | 5 | Missense | [E5,E80,E83,E95,E109] |
| c.781delinsCCATA | p.(Asp261Profs*2) | 1 | 0 | Frameshift | [E97] |
| c.985G>A | p.(Asp329Asn) | 1 | 0 | Missense | [E85] |
| c.336C>G | p.(His112Gln) | 1 | 11 | Missense | [1,97,E6,E31,E66,E80,E87,E110] |
| c.272A>G | p.(His91Arg) | 1 | 0 | Missense | [E5] |
| c.1346_1347insTT | p.(Lys449Asnfs*2) | 1 | 0 | Frameshift | [E27] |
| c.578C>T | p.(Pro193Leu) | 1 | 1 | Missense | [16,E21] |
| c.1226C>A | p.(Pro409His) | 1 | 0 | Missense | [E7] |
| c.794C>G | p.(Ser265*) | 1 | 0 | Nonsense | [E111] |
| c.950delC | p.(Thr317Argfs*25) | 1 | 0 | Frameshift | [E112] |
| c.660C>A | p.(Tyr220*) | 1 | 0 | Nonsense | [E80] |
| c.680_681delAT | p.(Tyr227Cysfs*27) | 1 | 0 | Frameshift | [20] |
| c.1445A>G | p.(Tyr482Cys) | 1 | 0 | Missense | [20] |
|  | p.(Val252Glyfs*11) | 1 | 0 | Frameshift | [E113] |
| c.754-?_1081+? | p.(Val252Thrfs*7) | 1 | 0 | Frameshift | [E48] |
| exon 7 duplication |  | 1 | 0 | Duplication | [E42] |
| 22q11.1 duplication |  | 1 | 0 | Duplication | [E32] |
| duplication of a region comprising exon 7 |  | 1 | 0 | Duplication | [E87] |
| c.542+1G>A | p? | 0 | 1 | Splice site | [97] |
| c.(972+1_973-1)_(1081+1_1082-1)del | p? | 0 | 1 | Splice site | [99] |
| c.-47+2T>C | p.? | 0 | 5 | Splice site | [E42,E87] |
| c.2T>C | p.? | 0 | 7 | Start loss | [E5,E67,E87] |
| c.753+2T>A | p.? | 0 | 5 | Splice site | [9,E1,E60,E98] |
| c.-2484del | p.? | 0 | 2 | Deletion | [E40] |
| c.972+3A>G | p.? | 0 | 1 | Splice site | [97] |
| c.973-149_1081+323del | p.? | 0 | 1 | Deletion | [E66] |
| c.326C>A | p.(Ala109Asp) | 0 | 2 | Missense | [1,E31] |
|  | p.(Ala357Thr) | 0 | 1 | Missense | [E5] |
| c.744delG | p.(Arg248fs) | 0 | 2 | Frameshift | [13,E114] |
| c.934C>T | p.(Arg312*) | 0 | 5 | Nonsense | [10,E32,E115] |
| c.100C>T | p.(Arg34Trp) | 0 | 3 | Missense | [E31,E85,E87] |
| c.133C>T | p.(Arg45Trp) | 0 | 1 | Missense | [E96] |
| c.144dupG | p.(Arg49Alafs*13) | 0 | 2 | Frameshift | [13,E80] |
|  | p.(Arg49Glyfs*4) | 0 | 2 | Frameshift | [E5,E67] |
| c.25C>T | p.(Arg9Trp) | 0 | 1 | Missense | [E37] |
| c.380A>T | p.(Asn127Ile) | 0 | 1 | Missense | [E38] |
| c.1269C>G | p.(Asn423Lys) | 0 | 4 | Missense | [9,E59] |
| c.158del | p.(Asn53Thrfs*12) | 0 | 1 | Frameshift | [24] |
| c.712G>A | p.(Asp238Asn) | 0 | 1 | Missense | [99] |
| c.1360G>C | p.(Asp454His) | 0 | 1 | Missense | [20] |
| c.1223G>C | p.(Cys408Ser) | 0 | 1 | Missense | [E94] |
| c.1223G>A | p.(Cys408Tyr) | 0 | 3 | Missense | [97,E24] |
| c.571delC | p.(Gln191Serfs*5) | 0 | 1 | Frameshift | [E94] |
| c.709delG | p.(Glu237fs) | 0 | 1 | Deletion | [E115] |
| c.731A>C | p.(Glu244Ala) | 0 | 1 | Missense | [E85] |
| c.984G>C | p.(Glu328Asp) | 0 | 7 | Missense | [10,13,E32,E116] |
| c.962G>C | p.(Gly321Ala) | 0 | 1 | Missense | [9] |
| c.977G>T | p.(Gly326Val) | 0 | 1 | Missense | [E38] |
| c.1148G>A | p.(Gly383Asp) | 0 | 2 | Missense | [E117] |
| c.1147G>A | p.(Gly383Ser) | 0 | 7 | Missense | [E39,E55] |
| c.1348G>T | p.(Gly450Cys) | 0 | 1 | Missense | [99] |
| c.142G>A | p.(Gly48Arg) | 0 | 3 | Missense | [E30,E38] |
| c.138_144del | p.(Gly48Trpfs*3) | 0 | 1 | Frameshift | [10] |
| c.13G>C | p.(Gly5Arg) | 0 | 1 | Missense | [E38] |
| c.334C>T | p.(His112Tyr) | 0 | 1 | Missense | [9] |
| c.396_397del | p.(His133Leufs*44) | 0 | 1 | Frameshift | [E118] |
| c.656A>C | p.(His219Pro) | 0 | 1 | Missense | [E60] |
| c.1004A>C | p.(His293Pro) | 0 | 2 | Missense | [E38] |
| c.427del | p.(Ile143Serfs*41) | 0 | 1 | Frameshift | [99] |
| c.629delT | p.(Ile210Thrfs*57) | 0 | 1 | Frameshift | [20] |
| c.278T>C | p.(Ile93Thr) | 0 | 5 | Missense | [1,9,13,E87,E114] |
| c.563T>C | p.(Leu188Pro) | 0 | 13 | Missense | [98,E3,E12,E66,E117-E119] |
| c.562C>G | p.(Leu188Val) | 0 | 2 | Missense | [9,E1] |
| c.746T>C | p.(Leu249Pro) | 0 | 5 | Missense | [10,E32] |
| c.932T>G | p.(Leu311Arg) | 0 | 3 | Missense | [8,E66,E88] |
|  | p.(Leu417Thrfs*4) | 0 | 1 | Frameshift | [E11] |
| c.274C>G | p.(Leu92Val) | 0 | 1 | Missense | [13] |
| c.163_165del | p.(Lys55del) | 0 | 2 | Deletion | [E109] |
| c.728T>G | p.(Met243Arg) | 0 | 4 | Missense | [97,E52] |
| c.927G>A | p.(Met309Ile) | 0 | 2 | Missense | [E5] |
|  | p.(Met71del) | 0 | 1 | Deletion | [E5] |
| c.620T>C | p.(Phe207Ser) | 0 | 1 | Missense | [20] |
| c.634_636delTTC | p.(Phe212del) | 0 | 3 | Deletion | [E5,E27,E36] |
| c.1065C>A | p.(Phe355Leu) | 0 | 2 | Missense | [13,E106] |
| c.1337T>C | p.(Phe446Ser) | 0 | 1 | Missense | [E43] |
| c.753G>A | p.(Pro251Pro) | 0 | 3 | Splice site | [9,13,99] |
| c.1031C>T | p.(Pro344Leu) | 0 | 3 | Missense | [10,E32] |
| c.1225C>T | p.(Pro409Ser) | 0 | 2 | Missense | [E5,E36] |
| c.1273C>G | p.(Pro425Ala) | 0 | 1 | Missense | [E85] |
| c.1303C>G | p.(Pro435Ala) | 0 | 2 | Missense | [E31,E85] |
| c.872C>T | p.(Ser291Leu) | 0 | 2 | Missense | [99,E94] |
| c.1435T>C | p.(Ser479Pro) | 0 | 4 | Missense | [E3,E32] |
| c.1448G>T | p.(Ser483Ile) | 0 | 1 | Missense | [E8] |
| c.385A>C | p.(Thr129Pro) | 0 | 3 | Missense | [E31,E53,E87] |
| c.559A>C | p.(Thr187Pro) | 0 | 1 | Missense | [E119] |
| c.97dupA | p.(Thr33Asnfs*) | 0 | 3 | Frameshift | [E85,E94] |
| c.1079C>T | p.(Thr360Ile) | 0 | 1 | Missense | [E8] |
| c.612G>C | p.(Trp204Cys) | 0 | 1 | Missense | [E31] |
|  | p.(Trp204Cys) | 0 | 1 | Missense | [E85] |
| c.791G>C | p.(Trp264Ser) | 0 | 2 | Missense | [2,E71] |
| c.718G>A | p.(Val240Met) | 0 | 2 | Missense | [E89] |
| c.1085G>A | p.(Trp362*) | 0 | 1 | Nonsense | [E3] |
| c.1196G>A | p.(Trp399*) | 0 | 1 | Nonsense | [E45] |
| c.1502G>A | p.(Trp501*) | 0 | 1 | Nonsense | [E85] |
| c.1501T>C | p.(Trp501Arg) | 0 | 2 | Missense | [98] |
| c.389A>G | p.(Tyr130Cys) | 0 | 1 | Missense | [E38] |
| c.680A>G | p.(Tyr227Cys) | 0 | 1 | Missense | [13] |
| c.706_708del | p.(Tyr236del) | 0 | 3 | Deletion | [13,E116] |
| c.1057T>C | p.(Tyr353His) | 0 | 2 | Missense | [E81] |
| c.452delC | p.(P151QfsX) | 2 | 0 | Frameshift | [E14] |
| c.973-?_1081+?del | p.(Val325Thrfs*7) | 0 | 2 | Frameshift | [E23] |
| c.973-314_1081+352del | p.(Val325Thrfs*7) | 0 | 1 | Frameshift | [E9] |
| c.1240G>A | p.(Val414Met) | 0 | 1 | Missense | [E43] |
| c.2T>A | p.Met1? | 0 | 1 | Start loss | [24] |
| c.1240_1442del |  | 0 | 2 | Exon deletion | [E86] |
| c.IVS6_IVS7del |  | 0 | 2 | Exon deletion | [E3] |
| c.956_1113del | p.(Asp319Glyfs*6) | 0 | 1 | Deletion | [E110] |
| c.1240_1420del |  | 0 | 1 | Deletion | [E33] |
| c.2475delC |  | 0 | 1 | Deletion | [E5] |
| c.114delG |  | 0 | 2 | Deletion | [99,E5] |
| deletion in exon 3 |  | 0 | 1 | Deletion | [E53] |
| deletion of exons 2–6 and 8–10 |  | 0 | 1 | Deletion | [96] |
| deletion of exons 8, 9, and 10 |  | 0 | 1 | Deletion | [E87] |
| intronic mutation |  | 0 | 1 | Intronic | [E50] |
